# Reclaiming human dignity: a critical review of contemporary theories in light of ontological foundations

**DOI:** 10.1007/s11019-025-10290-7

**Published:** 2025-08-02

**Authors:** Patrícia Frantz, Francisca Rego, Stela Barbas

**Affiliations:** Faculty of Medicine, Universidade do Porto, Porto, Portugal

**Keywords:** Human dignity, Ontology, Bioethics, Healthcare ethics, Metaphysical anthropology

## Abstract

Contemporary healthcare ethics often invokes the concept of human dignity as a normative cornerstone. Yet beneath this apparent consensus lies a fragmentation of meaning: dignity is variably interpreted as autonomy, capacity, recognition, or social construction—with little agreement on its essential content or justification. This conceptual disarray weakens the ethical coherence of bioethical decision-making and obscures the true nature of the human person. This article offers a critical review of the predominant contemporary theories of human dignity, including recognition-based approaches, capabilities theory, procedural pragmatism, and postmodern critiques. We expose the internal tensions and philosophical fragilities of each, especially when applied to medical practice. In contrast, we defend an ontologically grounded understanding of dignity—one that recognizes the human being as a unified, rational, embodied substance possessing intrinsic worth by virtue of being. By recovering this ontological foundation, we argue for a more coherent, universal, and morally resilient framework for healthcare ethics—one capable of upholding the inviolability of the person beyond shifting cultural, legal, or utilitarian paradigms.

## Introduction: the fragmentation of dignity

The concept of human dignity is one of the most frequently invoked—and least defined—principles in contemporary healthcare ethics. It appears in clinical guidelines, legal frameworks, international declarations, and academic discourse, often as a moral cornerstone. Yet beneath this apparent consensus lies a profound conceptual fragmentation. Dignity is alternately equated with autonomy, with cognitive capacity, with the capacity to flourish, or with social recognition. In many cases, it functions as a unifying term that accommodates a plurality of ethical languages but often lacks conceptual precision and coherence (Rosen and Dignity 2012). It is frequently treated as a flexible placeholder—a term of moral persuasion rather than one of philosophical clarity.

This multiplicity of meanings has consequences that are far from merely theoretical. In healthcare settings, where life, death, suffering, and vulnerability are at stake, the absence of a coherent and stable understanding of human dignity undermines ethical decision-making. It leaves room for arbitrariness, biases, and the quiet erosion of the very value it seeks to protect. When dignity is contingent on functional capacity or social validation, entire categories of patients—the elderly, the disabled, the unborn, the dying—are subtly, or explicitly, excluded from its full protection.

The aim of this article is to critically examine the major contemporary theories of human dignity—including recognition-based (Honneth 1995; Taylor 1994), capability-centered (Nussbaum 2006; Sen 1999), procedural (Dworkin 1993; Habermas 1996), and deconstructivist approaches (Angabem 1998; Butler 2004)—with particular attention to their influence on healthcare ethics. We argue that despite their valuable insights, these frameworks suffer from internal contradictions and philosophical fragility. More importantly, they fail to provide an ontologically grounded, universal account of what makes the human being worthy of respect, regardless of condition or context.

In contrast, we propose that human dignity must be reclaimed at its roots—in the very being of the human person. Only an ontological foundation, recognizing the person as a unified, rational, embodied substance with intrinsic worth, can provide the moral stability and universality that healthcare ethics requires. This approach does not aim to introduce a new theory, but rather to recover a deeper understanding of the person that can sustain the ethical demands placed upon health professionals.

## Recognition, capability, and constructivist paradigms: a critical survey

In recent decades, the discourse on human dignity has become increasingly diversified, reflecting a plurality of theoretical frameworks that attempt to articulate what it means to respect the human being. These frameworks often arise from commendable concerns—social injustice, marginalization, inequality—yet they frequently anchor dignity in extrinsic or relational criteria, thereby weakening its universality. Below we examine four prominent paradigms: recognition-based theories, the capabilities approach, procedural pragmatism, and postmodern deconstruction.

### Recognition-based theories

Rooted in social and political theory, recognition-based approaches argue that human dignity arises from the reciprocal acknowledgment of persons within social contexts. Thinkers such as Axel Honneth and Charles Taylor contend that identity and self-worth are shaped through processes of recognition—from intimate relationships to public institutions.

#### Strengths

This perspective rightly highlights the social dimension of human life and the psychological harm caused by misrecognition or exclusion.

#### Limitations

The central weakness of recognition theories lies in their dependence on external validation. If dignity depends on being recognized, then it is vulnerable to being denied. This undermines the very inviolability the concept is meant to protect. Moreover, such a framework fails to account for the dignity of those unable to participate in reciprocal recognition—the comatose patient, the unborn child, the person with severe cognitive impairment. The theory collapses in precisely the situations where the moral demand to affirm dignity is most urgent.

### The capabilities approach

Developed by Martha Nussbaum and Amartya Sen, the capabilities approach defines dignity in terms of the real opportunities available to individuals to develop and exercise essential human functions—from bodily integrity to practical reason, affiliation, and control over one’s environment.

#### Strengths

It moves beyond mere formal equality, focusing on what people are actually able to do and to be. It also emphasizes the role of social structures in enabling or disabling human flourishing.

#### Limitations

Despite its humanistic intention, the capabilities framework risks reducing dignity to a list of functional performances. It tends to marginalize those whose capacities are limited or undeveloped, such as individuals at the margins of life—the elderly with advanced dementia, the disabled, or the unborn. In practice, this leads to a conditional view of dignity, anchored not in being but in doing. As a result, dignity becomes a variable, rather than an inherent, attribute.

### Procedural pragmatism and legal positivism

Some contemporary theories, especially in juridical and political contexts, treat dignity as a procedural value—a kind of placeholder for moral consensus. Thinkers like Ronald Dworkin and Jürgen Habermas propose that dignity is best understood through participatory dialogue, constitutional interpretation, and the deliberative processes of democratic societies.

#### Strengths

Such approaches emphasize pluralism and the importance of legal frameworks in securing civil respect and rights. They seek to avoid authoritarian impositions by anchoring moral norms in public reason.

#### Limitations

However, procedural approaches often strip dignity of substantive content. They presume that moral legitimacy can be achieved without a shared metaphysical^1^ anthropology. The result is a formalism incapable of resisting deeply unethical outcomes, provided they are legally or procedurally sanctioned. History has shown that legality is no guarantor of justice. Without an anchor in the nature of the person, dignity becomes malleable—subject to the tides of political or cultural consensus.

### Postmodern and deconstructivist critiques

Some theorists, notably in the postmodern tradition, question the very coherence of “human dignity” as a universal category. Figures like Giorgio Agamben and Judith Butler deconstruct the notion of a stable human subject, viewing it as a construct embedded in power relations, language games, and historical contingencies.

#### Strengths

These critiques expose how dignity can be co-opted by dominant narratives and challenge the complacency of universalist rhetoric.

#### Limitations

But in deconstructing the subject, they also dissolve the foundation for any non-arbitrary affirmation of worth. Without a stable human essence, moral language becomes self-referential or purely strategic. In healthcare, this opens the door to ethical nihilism: if there is no inherent human nature, then there is no ground on which to assert the inviolability of the vulnerable.

This brief survey reveals a shared limitation across otherwise diverse frameworks: the absence of an ontological grounding for the human person. Whether focused on recognition, capability, procedure, or critique, these theories tend to treat dignity as a product—socially constructed, functionally measured, or procedurally enacted—rather than as a reflection of intrinsic being. It is precisely this ontological deficit that the next sections aim to address.

## Conceptual consequences for healthcare ethics

The absence of a coherent, ontologically grounded notion of human dignity is not a merely academic problem. In clinical settings, this theoretical deficit manifests as ethical confusion, inconsistency, and in some cases, as dehumanization. When dignity is redefined as functional ability, autonomous decision-making, or societal recognition, the scope of who is considered “fully human” narrows—often subtly, but with profound implications.

### Dignity in end-of-life decisions

In debates surrounding end-of-life care, the term “death with dignity” is frequently employed to justify practices such as physician-assisted suicide or euthanasia. Here, dignity is often reduced to autonomy—the capacity to make decisions about one’s own life and death. While autonomy is a real good, its elevation to the defining criterion of dignity results in a deeply problematic inversion: the person whose autonomy is impaired is seen as having lost their dignity, and thus becomes eligible for termination.

This view fails to recognize that dignity is not diminished by suffering or dependency, nor by the loss of self-determination. On the contrary, the final stages of life often reveal the depth of human worth in a more radical way—through vulnerability, relational interdependence, and existential confrontation. A dignity based on being, not doing, affirms the sacredness of life even in its most fragile moments.

### Dignity and disability

In contexts of disability, dignity theories based on capability or social contribution struggle to uphold equal moral worth. Individuals with severe cognitive or physical impairments may not meet the thresholds defined by capabilities theory or recognition frameworks, especially when these are interpreted through able-bodied norms (Taylor 1994; Nussbaum 2006).

This has led, in some cases, to the medical neglect or even active withdrawal of care from disabled individuals—not because of clinical futility, but because of perceived lack of “quality of life.” The silent logic at play is utilitarian: if dignity is measured by functioning or autonomy, then those who lack these are, implicitly, less worthy.

An ontological view of the human person resists this reductionism. It affirms that every person, regardless of capacity or consciousness, possesses dignity by virtue of being—not by fulfilling externally imposed criteria.

### Dignity in the beginnings of life

Similar distortions appear in the treatment of human life at its earliest stages. In reproductive technologies, genetic screening, and embryonic research, human embryos are frequently regarded as “pre-personal,” lacking full dignity until they acquire certain features—neurological development, sentience, or self-awareness (Meilaender 2009). This move severs the link between human nature and human worth.

The result is a selective ethic, in which some lives are welcomed and others are discarded based on their potential utility or desirability. The notion of “conditional dignity” here mirrors broader social attitudes toward the vulnerable: where ontology is denied, arbitrariness prevails.

These examples illustrate a common thread: when dignity is detached from what grounds the inherent worth of the human being, it becomes malleable—shaped by functional, legal, or social criteria. In such a climate, ethical judgments lose their stability, and the weakest pay the highest price.

In the next section, we argue that only a return to an ontological understanding of the person can restore coherence, universality, and moral depth to the concept of dignity—and with it, to the very heart of clinical ethics.

## The ontological foundation of human dignity

In contrast to contemporary theories that root human dignity in autonomy, function, or recognition, we propose that dignity is grounded in the ontological structure of the human being. This is not a romantic assertion, nor an appeal to religious sentiment. It is a metaphysical claim: dignity arises from what the human person is, not from what the person does, feels, or achieves. The conceptual fluidity surrounding the term—which today is invoked across disciplines without shared content—has obscured its foundations and weakened its moral force^2^. Restoring clarity requires returning to the being of the human person as the source of dignity.

Some contemporary thinkers have attempted to recover a universal meaning for dignity by emphasizing the moral singularity of each individual. One example is the political theorist George Kateb, who sees dignity as grounded in the “irreplaceability” of every person and in the human capacity for moral imagination. For Kateb, dignity is not derivative of community or religion but of the individual’s status as a moral being who can perceive and respond to the world in a uniquely human way (Kateb 2011). While this approach affirms intrinsic value, it remains conceptually dependent on subjective moral autonomy, rather than on a shared ontological structure.

### The human being as a unified substance

The human being is not a collection of parts or a temporary aggregation of functions. Rather, the person is a unified, living substance, composed of body and soul, matter and form, integrated into a single reality. This unity is not accidental, but essential. To be human is to be a rational and embodied subject—not merely conscious, but capable of truth, love, and moral responsibility.

This ontological integrity precedes any social recognition or functional capacity. It means that the human being is a bearer of value by nature, not by convention. Dignity, then, is not conferred. It is not earned. It is not subject to withdrawal. It is simply present—in every human life, from conception to natural death.

### Rationality and interiority

What distinguishes the human person from all other beings is the capacity to transcend mere instinct or reaction. The person is capable of interiority—of reflecting, choosing, and orienting their life toward ends that are not merely biological. This interiority, rooted in reason and will, is the formal principle of human dignity (Crosby 1996).

Even when obscured by illness or immaturity, this rational nature remains. A sleeping person, a fetus, or a patient in a coma does not cease to be human, because their nature is intact—even if their faculties are momentarily inoperative. Dignity is not a measure of actualization, but of essence.

### Embodiment and relationality

Human dignity is not opposed to vulnerability or limitation. In fact, embodiment is essential to personhood. The human person is not a mind trapped in a body, but a body-soul unity (Spaemann and Persons 2006). This embodied existence means that the human condition includes dependency, suffering, aging, and death—not as accidents to be eliminated, but as dimensions of a life lived in time.

Moreover, this embodiment implies relationality. No one comes into the world or survives alone. The person is always embedded in a web of relations—familial, communal, existential. To care for the sick, the weak, or the unborn is not an act of generosity toward an inferior being, but a recognition of shared nature and destiny.

### Ontology resists reduction

When dignity is anchored in the very being of the human person, it offers what contemporary theories often lack: stability. The human person remains the same in value whether strong or weak, young or old, conscious or unconscious. The dignity of a person with Alzheimer’s is equal to that of a healthy adult, not because of what they can do, but because of who—and what—they are.

This ontological account offers a framework capable of resisting the pressures of utilitarianism, legal positivism, and cultural relativism. It restores moral clarity where ambiguity now reigns, and it protects the vulnerable not through sentiment, but by appealing to the reality of their intrinsic worth.

Nonetheless, the persistence of “dignity” in legal, bioethical, and political discourses—despite its conceptual opacity—signals its normative appeal. As Christopher McCrudden has shown in his survey of the use of dignity in international law, the term functions as a placeholder for moral consensus across divergent traditions. Its utility lies precisely in its ambiguity, which allows for convergence without philosophical agreement (McCrudden 2008). Yet, this pragmatic function cannot substitute for a coherent account of*why* all human beings possess worth—a question that demands ontological depth.

This perspective aligns with the foundational language of international human rights instruments. The Universal Declaration of Human Rights (1948) opens with the affirmation of “the inherent dignity and the equal and inalienable rights of all members of the human family,” and Article 1 declares: “All human beings are born free and equal in dignity and rights” (General 1993). These formulations reflect an intuition that dignity precedes legal status or functional capacity—it is not granted, but recognized. However, the philosophical grounding of such dignity remains contested, and without a robust foundation, these affirmations risk becoming rhetorical rather than normative.

In the following section, we explore how this account of dignity not only withstands theoretical scrutiny, but also offers a reliable compass for ethical decision-making in healthcare—where the stakes are human lives.

## Ontological integrity and clinical application

A concept of dignity rooted in the ontological reality of the human person is not an abstract or impractical ideal. On the contrary, it offers the most coherent and resilient foundation for clinical ethics—one that resists the shifting tides of cultural norms, technological capabilities, or institutional pressures. When we understand the person as a rational, embodied substance with intrinsic worth, every aspect of healthcare is reoriented toward respect for being, not just for choice, performance, or prognosis.

### From patient to person

In many modern healthcare systems, the patient is implicitly reduced to a case, a pathology, or a bearer of symptoms. The ontological view restores the person to the center. A person is not defined by illness, diagnosis, or capacity, but by their unrepeatable existence (MacIntyre 1999). This shift transforms the clinical encounter: it is no longer a transaction of services, but a moral relationship between persons.

The healthcare professional, under this light, becomes not merely a provider of solutions but a custodian of human meaning. The act of care is not simply functional—it is ethical, even contemplative. It recognizes that healing is not always curing, and that presence, fidelity, and compassion can be modes of affirming dignity where medicine cannot eliminate suffering.

### Ethical coherence in vulnerable cases

Difficult clinical cases—end-of-life care, neonatal treatment limits, disability, mental illness—often strain ethical decision-making. The temptation in such cases is to rely on subjective assessments of “quality of life” or projections of future autonomy. But these criteria are unstable and often discriminatory.

The ontological approach, by contrast, provides a non-arbitrary criterion: if the being is human, the dignity is intact. This eliminates moral confusion and reorients clinical decisions around the inherent worth of the person, not their perceived utility, awareness, or potential.

For example:

- A comatose patient is not a “vegetative state,” but a person whose current incapacity does not erase their value.
- A child born with severe disabilities is not a “life unworthy of life,” but a bearer of the same dignity as any other child.
- A dying patient is not a burden to be managed, but a person to be accompanied to the threshold of eternity with reverence and care (Sulmasy 2007).

### Restoring the vocation of medicine

Finally, grounding ethics in ontology restores a vision of medicine as a moral vocation, not merely a technical enterprise. The physician is not a neutral executor of patient preferences, nor an engineer of outcomes, but a servant of the person (Pellegrino and Thomasma 1993). Clinical judgment, in this context, must be guided not only by empirical data but by a deep understanding of what it means to be human.

This does not negate the importance of autonomy, competence, or consent—but it places them in a hierarchy. The right to choose must be guided by the truth of who the chooser is. Freedom, separated from truth, becomes self-destructive. True care means guiding patients—even gently opposing them, when necessary—toward choices that honor their own nature.

An ontologically grounded ethics of dignity, therefore, is not a return to paternalism, but a movement toward moral realism: the courage to act in fidelity to the truth of the human person, even when such fidelity costs something.

In the final section, we will draw together the philosophical and clinical threads of this argument, and reaffirm the urgency of recovering the meaning of the human person in a time of ethical disorientation.

## Conclusion: recovering the meaning of the human person

In an age marked by rapid technological advances, moral pluralism, and institutional uncertainty, the concept of human dignity has become a frequent reference point—yet one increasingly emptied of stable meaning. Contemporary theories, though often well-intentioned, fail to provide a coherent and universal foundation for the value of human life. Whether grounded in autonomy, functional capacity, recognition, or procedural consensus, these frameworks lack the grounding necessary to uphold moral clarity in the face of real human vulnerability.

This article has argued that only a return to the ontological foundation of the human person can restore coherence and integrity to the discourse on dignity. The human being is not a construct, not a bundle of faculties or social relations, but a unified, rational, embodied substance—possessing dignity simply by being what it is. This truth does not change with age, ability, consciousness, or circumstance.

Such a foundation does not belong to any one ideology, religion, or cultural system. It arises from the very structure of reality. It demands no sentimentality, no ideological allegiance—only fidelity to the truth of what the human person is.

In healthcare, this truth has profound implications. It elevates the clinical encounter from a technical act to a moral relationship. It protects the weakest not by invoking compassion alone, but by recognizing that their being demands reverence. It reorients medicine toward its original vocation: the care of the person in their full, embodied humanity.

To recover the meaning of human dignity is, ultimately, to recover the meaning of the human person. And to do so is not a luxury—it is a necessity. For where the truth of the person is denied, ethics becomes arbitrary, and medicine itself risks becoming inhuman.

This is the task before us: not to invent new values, but to stand firm in what is already true—to defend, with quiet strength and intellectual rigor, the dignity that is not granted by society, but given by being.

## Footnotes

1 In this article, the term “metaphysical” is employed in its classical philosophical sense, referring to the inquiry into first principles and the foundational structure of being — not in the colloquial or mystical sense often attributed to it in contemporary usage.

2 Jean-Pierre Dupuy (2013) warns that the concept of dignity, when evacuated from its sacred or ontological background, becomes susceptible to arbitrary appropriation. See *The Mark of the Sacred* (Dupuy (2013).
