# Correction: Reclaiming human dignity: a critical review of contemporary theories in light of ontological foundations

**DOI:** 10.1007/s11019-025-10295-2

**Published:** 2025-09-02

**Authors:** Patrícia Frantz, Francisca Rego, Stela Barbas

**Affiliations:** Faculty of Medicine, Universidade do Porto, Porto, Portugal

**Correction to: Medicine, Health Care and Philosophy**

10.1007/s11019-025-10290-7

In this article, the order in which the authors appeared in the author list was incorrectly given as “Francisca Rego, Stela Barbas, Patrícia Frantz” where it should have been “Patrícia Frantz, Francisca Rego, Stela Barbas”.

The original article has been corrected.

